# Mpox (formerly monkeypox): pathogenesis, prevention and treatment

**DOI:** 10.1038/s41392-023-01675-2

**Published:** 2023-12-27

**Authors:** Junjie Lu, Hui Xing, Chunhua Wang, Mengjun Tang, Changcheng Wu, Fan Ye, Lijuan Yin, Yang Yang, Wenjie Tan, Liang Shen

**Affiliations:** 1 Xiangyang Central Hospital, Affiliated Hospital of Hubei University of Arts and Science, Hubei Province Xiangyang, 441021 China; 2 NHC Key Laboratory of Biosafety, National Institute for Viral Disease Control and Prevention, Chinese Center for Disease Control and Prevention, Beijing, 102206 China; 3 College of Biotechnology, Tianjin University of Science & Technology, Tianjin, 300457 China; 4 Shenzhen Key Laboratory of Pathogen and Immunity, National Clinical Research Center for infectious disease, State Key Discipline of Infectious Disease, Shenzhen Third People’s Hospital, Second Hospital Affiliated to Southern University of Science and Technology, Shenzhen, 518112 China

**Keywords:** Drug discovery, Infectious diseases

## Abstract

In 2022, a global outbreak of Mpox (formerly monkeypox) occurred in various countries across Europe and America and rapidly spread to more than 100 countries and regions. The World Health Organization declared the outbreak to be a public health emergency of international concern due to the rapid spread of the Mpox virus. Consequently, nations intensified their efforts to explore treatment strategies aimed at combating the infection and its dissemination. Nevertheless, the available therapeutic options for Mpox virus infection remain limited. So far, only a few numbers of antiviral compounds have been approved by regulatory authorities. Given the high mutability of the Mpox virus, certain mutant strains have shown resistance to existing pharmaceutical interventions. This highlights the urgent need to develop novel antiviral drugs that can combat both drug resistance and the potential threat of bioterrorism. Currently, there is a lack of comprehensive literature on the pathophysiology and treatment of Mpox. To address this issue, we conducted a review covering the physiological and pathological processes of Mpox infection, summarizing the latest progress of anti-Mpox drugs. Our analysis encompasses approved drugs currently employed in clinical settings, as well as newly identified small-molecule compounds and antibody drugs displaying potential antiviral efficacy against Mpox. Furthermore, we have gained valuable insights from the process of Mpox drug development, including strategies for repurposing drugs, the discovery of drug targets driven by artificial intelligence, and preclinical drug development. The purpose of this review is to provide readers with a comprehensive overview of the current knowledge on Mpox.

## Introduction

**Fig. 1:**
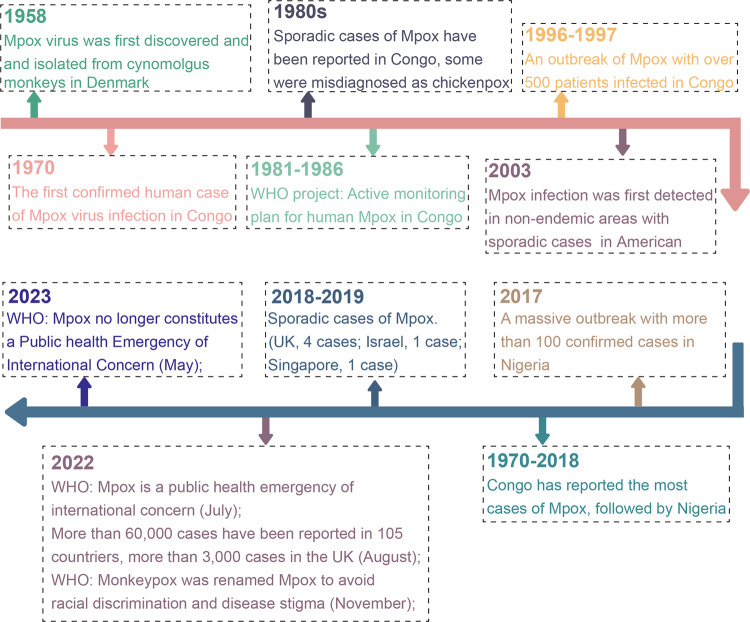
The timeline of the historical review and major milestones in Mpox

Mpox (formerly monkeypox) is an emerging zoonotic disease caused by Mpox virus infection, which affects both humans and animals.^1,2^ The virus was first discovered in monkeys in 1958 and has since been detected in a variety of animal species.^3^ The first human case of Mpox infection was diagnosed in 1970 in the Republic of the Congo, located in Central Africa.^4–6^ Subsequently, Mpox has predominantly circulated in Central and West Africa, with transmission occurring between animals (primarily primates and rodents), as well as between animals and humans, and through human-to-human contact.^7–9^ In recent years, the rapid globalization, population movement, and deepening trade networks have contributed to the international dissemination of Mpox, resulting in outbreaks in various countries worldwide.^10–12^ Notably, in 2022, a global outbreak of Mpox affected 110 countries and regions.^13^ Although the World Health Organization declared that Mpox outbreaks no longer constituted an “a Public Health Emergency of International Concern in May 2023,”^14^ it is important to highlight that certain regions in Asia have experienced a rise in Mpox cases due to the virus’s rapid evolution and increased international travel (Fig. 1).^15–17^ Cases of Mpox virus infection have been reported in cities such as Beijing, Guangzhou, and Shenyang.^18^ With the continuous increase in the number of infected patients, Mpox, a disease that was previously neglected, has re-entered the public attention.^19^ To effectively combat the disease, a renewed comprehension of Mpox is necessary. This review presents a comprehensive overview of Mpox, including its transmission patterns, pathogenesis, genome organization, and antiviral drugs that have been studied for their activity against Mpox over the past few decades, both in vivo and in vitro. Additionally, it provides helpful insights for the prevention and control of worldwide Mpox outbreaks by summarizing the valuable experiences gained from the development of anti-Mpox strategies, such as drug repurposing, drug target discovery, and the identification of potential drug targets.

## Transmission

**Fig. 2:**
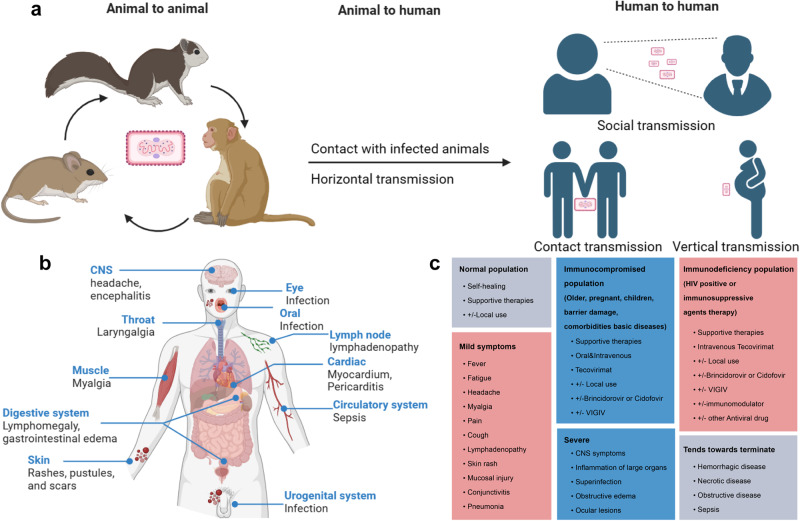
The epidemiological characteristics, pathogenesis, clinical diagnosis, and treatment of Mpox. **a** The transmission of Mpox occurs through animal-to-animal, animal-to-human, and human-to-human routes. **b** Clinical symptoms typically manifest after Mpox infection. **c** Symptoms of Mpox infection may vary based on the immune status and clinical treatment options and clinical treatment options are listed

The natural hosts of Mpox virus include some rodents and primates in central Africa. Early human infections are typically linked to contact with infected animals, including exposure to mucous membranes, body fluids, tissues, or consumption of undercooked meat. Transmission can also occur through scratches or bites from infected animals.^20^ Human-to-human transmission is believed to occur through direct contact with respiratory droplets from infected individuals.^21–23^ Furthermore, vertical transmission of Mpox virus can occur from infected mothers to their newborns. (Fig. 2a)^24,25^ This recent outbreak of Mpox infection was the largest reported epidemic outside of Africa, unlike previous outbreaks. In the past, Mpox infection was only diagnosed after contact with infected animals or traveling to regions affected by Mpox.^26–28^ However, in this current epidemic, most Mpox cases are not associated with contact with infected animals or travel, but with sexual contact between individuals.^29^ Over the past two years, the majority of reported cases of Mpox outbreaks have involved homosexual or bisexual males. A research study reported that 98% of cases were among homosexual or bisexual males, with 41% of them co-infected with human immunodeficiency virus (HIV). Additionally, 73% of the observed lesions occurred in the anal and genital regions.^30,31^ The incubation period of Mpox is ~7–14 days, with symptoms lasting for 14–21 days.^32,33^ The prolonged incubation period poses significant challenges for accurate diagnosis, potentially leading to delayed medical attention, disease progression, and further transmission of the virus.^34,35^

## Pathogenesis

Mpox is a self-limiting disease, and the severity of infection can be influenced by various factors, such as the specific viral strain, individual immune status, and potential complications that may arise.^36^ Common early symptoms of Mpox virus infection include pain, fever, fatigue, and lymphadenectasis, with significant inguinal lymphadenectasis often observed.^37–40^ The presence of lymphadenectasis can help to distinguish Mpox virus infection from other orthopoxviruses infection.^41^ Furthermore, understanding the transmission mode is essential in establishing effective measures to combat Mpox. Following exposure to the respiratory secretions or body fluids of Mpox patients, the Mpox virus enters nearby tissues through mucous membranes (such as ocular, respiratory, oral, urethral, and rectal) or broken skin.^42,43^ It then spreads throughout the body via tissue-resident immune cells and draining lymph nodes.^42,44^ This constitutes the latent period for Mpox virus infection, which typically lasts up to two weeks. Throughout this period, individuals infected with Mpox are generally asymptomatic and devoid of lesions. Following the latent period, individuals infected with Mpox virus begin to exhibit atypical symptoms, including fever and chills, headache, muscle pain, and lymphadenectasis. These initial prodromal symptoms of Mpox typically last for three days. After the fever and lymphadenectasis, rashes begin to appear on the head and face, and gradually spread throughout the body. The rash evolves from papules to vesicles and pustules, and ultimately forming crusts that heal, leaving behind scars. This progressive rash phase lasts about 2–4 weeks.^43,45,46^ In the current outbreak of Mpox among men who have sex with men (MSM), some unusual clinical signs have been observed with rashes appearing primarily around the genital or anal area and subsequently spreading throughout the body.^27,47^ Severe cases of Mpox virus infection can lead to complications such as hemorrhagic disease, necrotic disease, obstructive disease, inflammation of vital organs, and septicemia. The case fatality rate of Mpox in non-epidemic regions during 2022 was ~0.04%. (Fig. 2b)^3,25,38,48–50^ Immunocompromised individuals, including children, older adults, and those with immunodeficiencies (such as HIV patients and individuals using immunosuppressive medications), are more susceptible to experiencing these severe manifestations. In addition, immunocompromised individuals are more likely to contribute to the evolution of Mpox, making it increasingly adaptable to human hosts and resulting in widespread transmission (Fig. 2c).^46,51–54^

## Virus morphology and genome

**Fig. 3:**
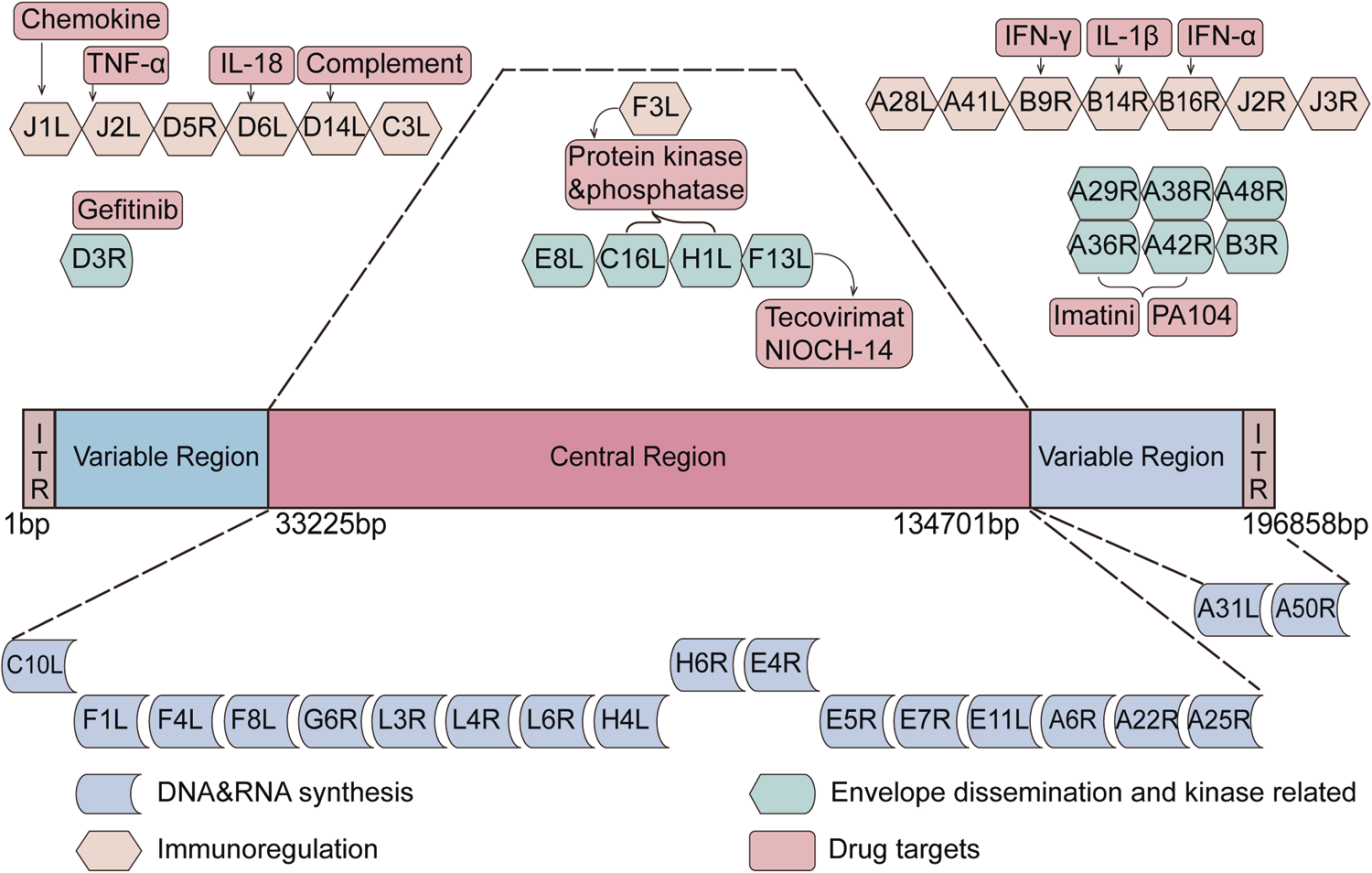
The genome structure and potential antiviral targets of Mpox virus. The Mpox virus genome consists of a double-stranded linear DNA comprising approximately 196,858 base pairs. It consists of a central recognition region, two variable region, and two terminal inverted terminal repeats (ITRs) (Monkeypox virus strain Zaire, GenBank accession number: AF380138.1, web link: https://www.ncbi.nlm.nih.gov/nuccore/17529780). In the genome map, target genes implicated in the interaction between Mpox virus and antiviral drugs are listed. Most essential genes are located in the central region of the genome

Mpox is caused by Mpox virus, a member of the genus *orthopoxvirus* in the family Poxviridae, is characterized by its brick-shaped or oval morphology with a diameter of ~200–250 nm.^55,56^ Its genome consists of a linear, double-stranded DNA with a length of ~197 kb and encoding about 180 proteins.^57^ Additionally, Mpox virus possesses dumbbell-shaped nucleocapsid enveloped by ovoid lipid-containing particles. The genomic structure of Mpox virus closely resembles that of other orthopoxviruses, characterized by a highly conserved central core region, variable regions at the left and right ends, and a tandemly repeated inverted terminal repeat. (Fig. 3)^58,59^ The central core region of Mpox virus shares more than 90% sequence homology with other orthopoxviruses, particularly within the open reading frame (ORF) located between C10L and A25R.^57,60^ Species and strain-specific characteristics of orthopoxviruses are often found in the variable regions at the ends of the genome. A better understanding about these ORFs may provide insights into its host tropism, pathogenesis, and differences in immune regulation.^28^ Based on a genomic and phylogenetic analysis conducted in 2022, the prevalent strain of Mpox virus was identified as belonging to the B.1 lineage of the West African clade. The B.1 lineage exhibits multiple mutations in genes associated with virulence, host recognition, and immune evasion.^61^ In comparison to previously obtained complete genome sequences of Mpox virus isolated in Nigeria from 2017 to 2018, the Mpox virus strains that emerged in 2022 exhibited a higher number of single nucleotide polymorphisms (SNPs). The Mpox virus strain isolated in 2022 exhibit ~50 SNPs, indicating an approximately 6–12-fold increase in the predicted substitution rate of Mpox virus compared to the strains isolated from 2018-2019 (1–2 nucleotide substitutions per genome every year).^62,63^ The functional significance of these mutations is yet to be fully understood, but this high mutation rate may help explain the sudden appearance and heightened transmissibility of Mpox virus in non-endemic regions.

## The life cycle of Mpox virus and the discovery of anti-Mpox virus drugs

The process of Mpox virus infection and replication can be summarized into three distinct stages: 1) virus invasion; 2) virus replication and synthesis; 3) virus assembly, maturation and release.^64–66^ Targeting any stage of the Mpox virus lifecycle holds promise for the development of effective antiviral interventions against Mpox virus.

### Anti-Mpox virus drugs targeting the invasive phase

**Fig. 4:**
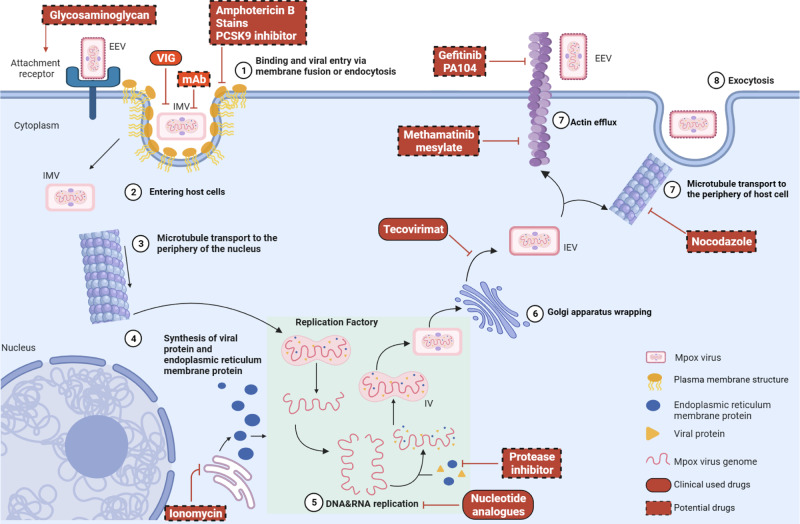
The life cycle of Mpox virus replication in hosts and potential targets for anti-Mpox virus drugs. The complete life cycle of Mpox virus infection: from entry into host cells to excretion. Briefly, both EEV and IMV viral particles penetrate the host membrane through membrane fusion and endocytosis. Mpox virus viral particles utilize glycosaminoglycans as host receptor. IMV particles enter the cytoplasm and are transported to the perinuclear replication factory via microtubules. The released Mpox virus genome serves as a template for DNA replication. Furthermore, IMV are enveloped by the Golgi apparatus to form IEV, and are transported to the cell surface via actin or microtubules. Part of the important drugs targeting each stage of the replication process are listed. EEV extracellular enveloped virions, IMV intracellular mature virions, IEV intracellular enveloped virions, IV immature virion

The development of antiviral drugs begins with a thorough understanding of the complete life cycle of the virus (Fig. 4). In the early stages of Mpox virus infection, two distinct infectious viral particles are present: extracellular enveloped virions (EEV) and intracellular mature virions (IMV).^67^ These viral particles vary in surface glycoprotein and envelope membrane composition, with IMV exhibiting a single-membrane structure and EEV possessing a double-membrane structure. IMV are released only upon cell lysis and enters host cells through direct fusion and endocytosis,^68,69^ while EEV enters via membrane fusion.^70–72^ IMV are the most abundant viral particles in terms of quantity, due to the absence of a lipid membrane, which gives them a simpler and more robust structure.^73^ This enhances their resistance to external damage, prolonging their survival time outside the host. However, the exposed surface proteins of IMV trigger higher production of neutralizing antibodies and activate complement responses.^74–76^ Additionally, these exposed surface proteins enhance the recognition and inactivation of Mpox virus by immune cells. In contrast, EEV possesses an additional lipid membrane layer on their surface, enabling better intracellular dissemination.^69^ The pox virus can utilize lipid rafts on the lipid membrane to enter host cells, and cholesterol is one of the important components responsible for maintain the structure and function of lipid rafts.^77,78^ Amphotericin B, a long-standing antibiotic used for the treatment of fungal infections, can sequester cholesterol within host cell membranes, disrupting the integrity of lipid raft and potentially inhibiting Mpox virus infection.^79^ Additionally, cholesterol-lowering drugs such as statins and PCSK9 inhibitors may exhibit antiviral activity by modulating cellular cholesterol levels.^80^ Mpox virus attaches to mucous membranes and damaged skin, where a high concentration of glycosaminoglycans (GAGs) are present. GAGs serve as primary attachment receptors for host cells. EEV particles of Mpox virus interact with GAGs and enter host cells. Marine sulfated polysaccharides are natural analogs of GAGs that competitively bind to the host cell membrane surface, thereby preventing the attachment and entry of Mpox virus.

Since no specific receptor for Mpox virus on the host cell membrane has been found so far, several envelope proteins that play a key role in the invasion of host cells by Mpox virus, may be attractive targets for the development of anti-Mpox virus drugs. He et al. evaluated the binding capacity of eight marine sulfated polysaccharides to the surface envelope protein A35R of Mpox virus using surface plasmon resonance technology. The research findings indicated that some sulfated polysaccharides exhibited competitive binding effects and anti-Mpox virus activity.^81^ In another study, Li et al. inoculated BALB/c mice with recombinant A35R protein and purified the A35R immune serum, which showed high neutralizing activity against two types of vaccinia virus (VACV)-EEV.^82^ Moreover, several IMV surface membrane proteins, including I5L, E8L, and A43R have been identified through whole-genome sequencing, and may facilitate the entry of Mpox virus into host cells through receptor and membrane fusion.^57,60^ Although the exact mechanisms of interaction between these proteins and the host are not fully understood, they could potentially serve as targets for future anti-Mpox virus discovery. Further research is needed to unravel the specific roles of these proteins in Mpox virus infection.

### Antiviral drugs that influence viral replication and synthesis

After IMV or EEV enter the host cell, the exposed viral core is transported to the periphery of the cell nucleus through microtubule structures at an average speed of 52 μm/min.^83^ The viral core consists of the central viral genome and an enveloped nucleocapsid. The mechanism of nucleocapsid uncoating involves ubiquitination of the nuclear capsid proteins and degradation by proteasomes.^84–86^ Once uncoating is completed, the Mpox virus genome begins efficient replication, rapidly amplifying like a factory.^87–91^

Currently, researchers are devoted to developing anti-Mpox drugs by interfering with the DNA or RNA synthesis of the viral genome.^92^ Nucleoside analogs are chemical compounds that have a similar structure to naturally occurring nucleosides. These drugs competitively bind to the viral DNA or RNA polymerase, disrupting the replication process by causing termination of the DNA or RNA chain synthesis. Due to their ability to inhibit viral replication, these drugs often exhibit broad-spectrum antiviral activity.^93,94^ Cidofovir, a non-cyclic monophosphate nucleoside analog, can be used for the treatment of orthopoxviruses and demonstrate potent antiviral activity in vitro (Mpox virus, effective concentration half maximal (EC_50_) = 2.52 μg/mL, Selectivity index (SI) = 15, in human embryonic lung fibroblasts) and in vivo (Mpox virus, 5 mg/kg, cynomolgus macaques, intraperitoneal injection; Mpox virus, 5 mg/kg, human, intravenous).^95–98^ Following the Mpox outbreak in 2022, Cidofovir was rapidly employed in clinical trials for the treatment of Mpox^99–101^ However, Cidofovir is a divalent anion with low bioavailability. In patients with impaired renal function or undergoing renal replacement therapy, its metabolites can accumulate in proximal renal tubular cells, leading to kidney damage.^102–104^ In order to overcome the limitations of Cidofovir, its derivative Brincidofovir has been developed. Brincidofovir has been modified using lipid conjugation technology, resulting in improved cellular uptake and conversion capabilities, it was approved by the FDA in 2021 for the treatment of smallpox.^105^ Unlike Cidofovir, Brincidofovir does not require metabolism through the renal anion transport system, thus exhibiting higher bioavailability and no significant nephrotoxicity in vitro (VACV, EC_50_ = 0.19 μM, in vero cells) and in vivo (Mpox virus, 10 mg/kg, mice, gastric gavage; Mpox virus, 200 mg, human, oral).^105–109^ However, Brincidofovir still presents some adverse reactions such as gastrointestinal reactions and liver function injury.^101,110^ Apart from Brincidofovir, other compounds based on structural modifications of Cidofovir have been developed.^111,112^ For instance, NPP-669 is synthesized by linking a long-chain sulfonate to Cidofovir. This modification improves its solubility in water and affinity for lipid through alkyl chain modification. As a result, this structural modification enhances the metabolic stability and bioavailability while reducing nephrotoxicity. It has shown enhanced antiviral effectiveness in vitro (vaccinia, EC_50_ = 8.95 μM, in HFF cells) and in vivo (cytomegalovirus, 3 mg/kg, mice, intraperitoneal injection).^113^ Ribavirin, a well-known nucleoside analog, blocks viral nucleotide synthesis and thus inhibits viral replication and transmission. It has broad-spectrum antiviral efficacy against various DNA and RNA viruses, including Mpox virus. Studies have shown that ribavirin can impede the replication of orthopoxviruses in vitro (Mpox virus, EC_50_ = 5.9 μg/mL, in Vero cells) and in vivo (cowpox virus, 50 mg/kg, mice, subcutaneous injection).^114,115^ However, further clinical studies are needed to assess its effectiveness in Mpox patients are needed. Although nucleotide analogs possess potent antiviral effects, they also have the potential to induce viral resistance. Recently, resistant Mpox virus strains to Cidofovir have been identified.^116–118^ Consequently, researchers are currently focused on the development of new nucleotide analogs such as KAY-2–41, a novel guanosine analog developed by Sophie et al. This analog exhibits potent antiviral activity against VACV in vitro (VACV-WR, EC_50_ = 0.8 μM, SI = 18, in human embryonic lung cells) and in vivo (VACV-WR, 50 mg/kg, mice, intraperitoneal injection), remaining effective against Cidofovir-resistant strains.^119,120^ This discovery provides a new therapeutic approach for nucleotide-resistant Mpox virus strains that are currently in use. However, further research and evaluation are needed for the clinical application of this novel nucleotide analog. The DNA-dependent RNA polymerase (DdRp) plays a crucial role in catalyzing the replication process of DNA viruses in the cytoplasm. Due to its biological significance, DdRp is considered a potential therapeutic target for Mpox virus. Through computer modeling of DdRp, along with techniques such as molecular dynamics simulations, docking, and computational screening, potential inhibitors of DdRp can be efficiently identified. Several small molecule compounds with inhibitory activity against DdRp have been discovered through computer-assisted drug design.^121–123^ However, further researches are needed to validate these identified candidate compounds, evaluate their safety and effectiveness, and ultimately progress them to the clinical application stage. The endoplasmic reticulum (ER) plays a crucial role in enveloping and stabilizing the viral genome. Electron microscopy observations have shown that the replication factory is surrounded by a significant amount of ER membrane. The ER plays a key role in the synthesis of viral membrane proteins.^124,125^ Subsequently, these membrane proteins, together with other viral structure proteins, enter into the viral factory, encapsulate the core genes, forming crescent-shaped structures.^125,126^ Moreover, the presence of the ER is important for maintaining the stability of the viral genome. Studies have indicated that ionomycin disrupts the integrity of ER in vitro, resulting in the inability of ER membrane proteins to enclose the exposed genome. This exposure triggers an immune response, leading to the degradation of viral DNA and significantly impacting VACV DNA replication.^127^ This discovery highlights the essential role of the ER in maintaining Mpox virus genome stability and facilitating viral replication. Based on these findings, compounds that effectively inhibit the formation of ER membrane proteins could also serve as potential antiviral drugs against Mpox virus.

### Antiviral drugs that affect virus assembly, maturation, and release

Within the replication factory, these crescent-shaped structures develop into ellipsoidal or spherical shapes, representing immature virion (IV) particles.^56,127^ IV particles undergo the proteolytic cleavage of several capsid proteins and the condensation of the core, resulting in the formation of mature virus particles known as IMV. These IMV are abundant within the host cells. As IMV proliferate, they cause the lysis of the host cells, subsequently releasing IMV viral particles. Additionally, a portion of the IMV exits the virus factory through the microtubule organizing center and becomes enveloped by the trans-Golgi network (TGN) or the nuclear membranes, forming intracellular enveloped virus (IEV).^128–131^ Compared to the single-layered membrane structure of IMV, IEV possesses a three-layered membrane structure. During the early stage of infection, a majority of IMV are enveloped to form IEV. However, in the later stages of infection, IMV become the predominant form, possibly due to the depletion of TGN and nuclear membranes.^132,133^ Once IEV reach the peripheral region of the cell, the viral envelope fuses with the host cell membrane, forming cell-associated enveloped viruses (CEV) through the process of exocytosis.^134^ Virus particles remaining on the surface of the host cell are referred to as CEV, whereas those released into the extracellular environment are referred to as EEV.^135^ The ratio of EEV to CEV depends on the specific virus strain and host cell type. While the mechanism by which EEV released from infected cells further infect neighboring cells is not fully understood, researchers have discovered that the actin tails can form and extend a long distance outside the cell. EEV can utilize actin tails to enter adjacent cells, establishing bridges between the actin tails and neighboring cells, thereby facilitating efficient viral spread.^136–138^

In order to reach the cellular plasma membrane, the release of viruses requires the involvement of the actin cytoskeleton.^139^ Currently, two mechanisms have been proposed to explain how IEV traverse the actin cytoskeleton. The first mechanism is actin polymerization-induced assembly. Upon viral infection, host cells trigger the polymerization of actin, resulting in the formation of filamentous structures known as actin tails. Failure to form actin tails hinders virus migration, adhesion, and intercellular spread. Several studies suggest that tyrosine phosphorylation plays a pivotal role in the formation of actin tails.^140,141^ The second mechanism is microtubule transport. IEV reach the cell surface through microtubule-mediated transport. Studies conducted by Hollinshead et al. observed the movement trajectory of viral particles labeled with green fluorescent protein.^142^ They found that viral particles co-localized with microtubules and exhibited an average velocity of 60 μm/min, consistent with the speed of microtubule transport. This speed far exceeds the transport rate of actin tails (2.8 μm/min).^143,144^ The movement of viral particles to the cell surface can be hampered by the microtubule-depolymerizing drug nocodazole in vitro.^145–147^ Based on this evidence, it is evident that microtubule transport plays a crucial role in the externalization of IEV to the cell surface. Disruption of microtubule structures may contribute to reducing the export of virus particles and inhibiting the spread of infection. As we mentioned earlier, the actin tails play an important role in the process of Mpox virus infecting of neighboring cells. Several drugs have been reported to inhibit the formation of actin tails, including the anti-cancer drug imatinib mesylate, which has shown anti-*orthopoxvirus* activity in vitro.^148^ Furthermore, a compound named PA104, identified by Lalita., has been shown to significantly inhibit the formation of actin tails, thereby reducing viral release and spread in vitro (VACV, EC_50_ = 0.8 μM, SI > 800, in BSC40 cells).^149^ Epidermal growth factors (EGFs) encoded by orthopoxviruses, play a crucial role in intercellular virus transmission.^150,151^ EGF can activate the EGFR/MEK/FAK signaling pathway, promoting intercellular virus transmission and facilitating rapid movement of infected cells.^152^ This increases the likelihood of contact between infected and uninfected cells, ultimately enhancing the transmission efficiency of orthopoxviruses.^153,154^ Experimental findings demonstrate that the use of EGFR inhibitor gefitinib and MEK inhibitor effectively reduces the area of virus-infected plaques in vitro (VACV, EC_50_ = 4.93 μM, in Hep2 cells).^155^ Notably, gefitinib reduces actin tail formation by 1.6-fold and decreases infected cell migration efficiency by fourfold.^153^

The viral-encoded membrane proteins of orthopoxviruses also play a significant role in viral transmission. Research has demonstrated that the Mpox virus A36R protein is crucial role in intercellular virus transmission and the release of EEV into the surrounding environment.^156^ Mohammad et al. identified three peptides that effectively targeting A36R have been identified and exhibit effective antiviral activity against Mpox virus. These peptides show a high affinity for A36R while being non-allergenic and non-toxic.^157^ Furthermore, a study on vaccinia virus revealed that the absence of A33R and A34R proteins increases EEV production,^158^ while the absence of A36R and B5R proteins decreases EEV production.^159^ This suggests that Mpox virus-encoded proteins with homology with A36R or B5R could potentially be valuable antiviral targets for future therapeutic strategies against Mpox virus. The VP37 protein, encoded by the F13L gene, is a catalytic protein involved in the intracellular envelopment of mature viral particles.^160–162^ It is widely distributed and highly conserved among orthopoxviruses, playing a crucial role in the in the formation of IMV within the TGN.^160^ It is essential for the virus’s pathogenicity and infectivity. Deletion of the F13L gene hinders the membrane envelopment process of orthopoxviruses, limiting their further spread.^163^ Tecovirimat, initially named ST-246, is a compound discovered through high-throughput screening (HTS) that exhibits potent antiviral activity against Mpox virus in vitro (Mpox virus, EC_50_ = 0.01 μM, in Vero cells) and in vivo (Mpox virus, 10 mg/kg, cynomolgus macaques, gavage; Mpox virus, human, 600 mg bid, oral).^164–167^ Tecovirimat functions by inhibiting the synthesis of the VP37 protein, thereby impeding the maturation process of orthopoxviruses and disrupting their envelopment and release.^168–170^ Tecovirimat primarily restricts intercellular virus spread without affecting the viral replication process. It has been identified as one of the most effective drugs against orthopoxviruses. Following the Mpox outbreak in 2022, the U.S. Food and Drug Administration granted emergency approval for Tecovirimat as a therapeutic drug for Mpox, demonstrating its promising clinical efficacy.^171^ Between May 2022 and February 2023, Germany reported 12 severe cases of Mpox, in patients with either severe immunosuppression due to HIV infection (CD4 + T cell count below 200/μL) or significant systemic involvement (over 100 skin lesions). These patients underwent treatment with tecovirimat, and clinical outcomes revealed complete recovery in all patients, with Tecovirimat exhibiting excellent tolerability.^172^ In addition to Tecovirimat, another compound called NIOCH-14, serving as a precursor to Tecovirimat, has shown specific antiviral activity against orthopoxviruses and shares structural similarity with Tecovirimat in vitro (Mpox virus, EC_50_ = 0.013 μg/mL, in Vero cells) and in vitro (Mpox virus, 40 mg/kg, marmot, oral). Unlike tecovirimat, NIOCH-14 offers a simpler synthesis route, thereby reducing costs and technical requirements. This natural advantage makes NIOCH-14 promising candidate for future wide-ranging applications.^100,173–175^ The current round of Mpox outbreak is highly mutable and still evolving. Although Tecovirimat is effective in current clinical use, it may pose a risk of resistance in the future. Discovering drugs with a new mechanism of action could provide a solution to the drug resistance problem. PA104 suppressed the formation of extracellular virus particle and viral propagation by inhibiting actin tail formation. Its mechanism of action differed from that of Tecovirimat. Of note, PA104 has demonstrated the ability to inhibit the replication of Tecovirimat-resistant VACV strains in vitro.^149^

## Immunothreapy in Mpox

Mpox virus-induced immunopathology leads to adverse outcomes in clinical, and immunotherapy for Mpox has the potential to reduce severe cases. Antibody-based therapeutics, immune cell, Immune effector molecules, and Modulation of cellular signal transduction are potential immunotherapies. Combination antiviral drugs with immunotherapy may be more effective and provide greater clinical benefit than single antiviral therapy alone.^176–178^

### Immune globulin and antibodies

Antibody-based therapeutics have shown significant progress in treating certain infectious diseases and currently being actively explored.^176,179^ Immune globulin, convalescent plasma, and neutralizing antibodies offer promising options as adjunctive treatments for cases with insufficient antiviral drug efficacy in severe patients.^180–182^ Notably, individuals who have been previously vaccinated with the smallpox vaccine produce more neutralizing antibodies that may be cross-protective against Mpox virus infection.^183^ Thus, some countries have approved the intravenous administration of vaccinia immune globulin (VIGIV) for managing complications associated with smallpox vaccination.^184^ For individuals with severe T cell functional immunodeficiency due to contraindications to smallpox vaccination, VIGIV can be considered as a prophylactic measure in vitro (5 mg, incubated with VACV) and in vivo (VACV, 400 mg/kg, mice, intravenous) and in one clinical case (6000 U/kg, single-dose intravenous).^185,186^ Although convalescent plasma (CP) therapy shows therapeutic potential for other infectious viruses,^187,188^ there is currently no available literature regarding its use for the treatment of Mpox infection.^176^

Li et al. demonstrated that monoclonal antibodies (mAbs) targeting the specific proteins (A29L and A35R) of the Mpox virus effectively neutralized orthopoxviruses, including VACV. These mAbs also showed protective effect in mice, resulting in reduced viral titers and alleviation of lung injury.^82^ Gilchuk et al. identified a large number of *orthopoxvirus*-specific mAbs from the blood cells of human subjects with a history of prior orthopoxvirus vaccination or infection, and of which 16 mAbs had neutralizing activity against Mpox were identified. Moreover, mAbs targeting A33, L1, A27, or H3 antigens exhibited the broadest cross-neutralizing activity against VACV and Mpox virus. In vivo experiments confirmed that a combination of mAbs with high neutralizing activity provides efficient protection against lethal doses of VACV infection in mice (VACV, 1.2 mg mAbs, intraperitoneal injection), compared to VIGIV. This protective effect was observed even in severe combined immune-deficiency mouse models.^181^ Therefore, mAbs drugs are most likely to provide effective clinical treatment outcomes in the development of anti-Mpox treatments compared to VIGIV and CP, which have uncertain efficacy.

### Immune cells

Mpox virus enters the human body through mucous membranes or compromised skin, resulting in infection of resident immune cells and antigen-presenting cells in the tissues.^189–191^ Subsequently, Mpox virus rapidly replicates in draining lymph nodes and disseminates through the lymphatic system,^192,193^ explaining the characteristic lymph node enlargement observed in Mpox virus infections. Innate immune cells act as the first line of defense against viral infections and are primary targets for viral assault. During the early stages of Mpox virus infection, monocytes are recruited to the infection site and become early targets for viral infection.^194^ The level of Mpox virus antigens detected in monocytes can serve as an indicator of infection severity and prognosis. Additionally, natural killer (NK) cells play a crucial role in generating a robust immune response following Mpox virus infection. Despite an increase in the abundance of NK cells in Mpox virus-infected individuals, their migration, degranulation, and effector molecule release capabilities are significantly impaired. Patricia’s study demonstrated that the injection of in vitro expanded NK cells into mice infected with vaccinia virus resulted in significantly prolonged mouse survival and enhanced secretion of IFN-γ by NK cells.^195,196^ T cells, another vital immune cell type, possess cytotoxic functions and the ability to regulate disease severity. Individuals with acquired immunodeficiency are at high risk of severe infections when co-infected with Mpox virus, often requiring active medical intervention rather than spontaneous resolution.^197,198^ Furthermore, individuals with compromised immune function are more susceptible to severe disease and mortality during Mpox virus infection. Other immune cell types, such as dendritic cells and innate lymphoid cells, also undergo alterations during Mpox virus infection.^199^ Understanding the characteristics and transformations of diverse immune cells during Mpox virus infection is important for gaining insights into the immune response and developing immunotherapy.

### Immune effector molecules and immunomodulators

**Fig. 5:**
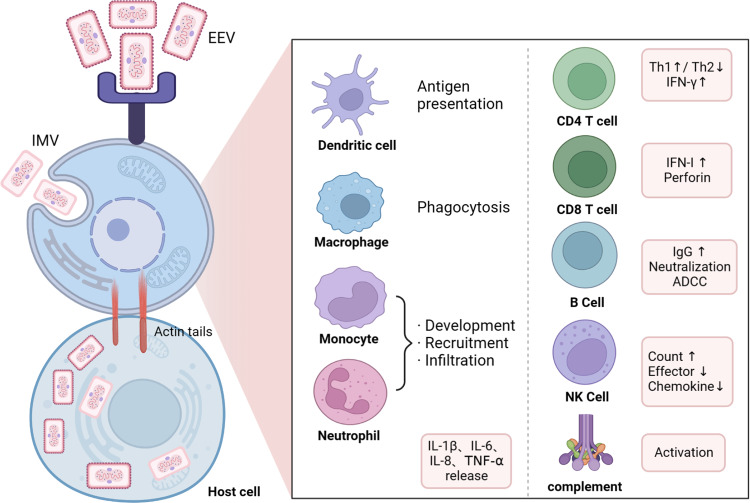
The host cell immune response after Mpox virus infection. The Mpox induces specific and non-specific immune responses after infection. Briefly, upon entry of Mpox virus into host cells, mononuclear phagocytes and neutrophils initiate recruitment and increased proliferative infiltration, other antigen-presenting cells (such as dendritic cell) become activated, leading to the release of effector molecules and chemokines, while other cells (T cells, B cells, NK cells and the complement system) of the immune system also begin to exert their corresponding effector functions. IL interleukin, Th helper T cell, IFN Interferon, ADCC antibody-dependent cell-mediated cytotoxicity

The response of immune effector molecules during Mpox virus infection—plays a crucial role in disease progression and severity. At the beginning of infection, the Mpox virus can suppress the expression of chemokines, resulting in a decrease in effector molecule expression like IFN-γ and TNF-α. This inhibition in T-cell activation hinders the initiation of humoral immune response, allowing the virus to evade the immune system much easier. However, severe Mpox infection often leads to a cytokine storm in later stages. This results in an increase in Th2-associated cytokines and a decrease in Th1-associated cytokines, characterized by increased expression of IL-2, IL-4, and IL-8 and a reduction in TNF-α, IL-2, and IL-12 (Fig. 5). By regulating these immune effectors, Mpox virus suppresses the antiviral immune response and disrupts the host immunity. Ribavirin, in addition to blocking viral nucleotide synthesis, also acts as an immunomodulator. It regulates T-cell polarization, and can enhance the release of interferon-gamma (IFN-γ) and T-bet, which are associated with Th1 response, in the serum of patients infected with hepatitis viruses. Simultaneously, ribavirin suppresses the release of GATA binding protein 3 and IL-4, which are related to Th2 response, promoting T-cell polarization towards Th1 and strengthening the antiviral action of the immune system.^200–202^ Ribavirin also stimulates the generation of central memory T-cells and Tregs.^203,204^ Pidotimod is an immunomodulator used as an adjunctive therapy for respiratory or urinary tract infections.^205^ It promotes non-specific and specific immune responses by activating NK cells, stimulating lymphocyte proliferation, and inducing the release of IL-1β and IFN-γ.^206–209^ Thymosin is an exogenous polypeptide with immunoregulatory effects that promote T cell differentiation, development, and maturation.^210,211^ Additionally, Thymosin can indirectly enhance the immune responses of other immune cells.^212,213^ Nevertheless, the clinical use of pidotimod and thymosin preparations in Mpox virus-infected patients has not been reported and requires further investigation to validate their efficacy. Although immunomodulators cannot directly elicit an anti-Mpox virus effect, they have the potential to improve the immune system, which may help reduce the development of severe manifestations and decrease mortality rates.^210,211,214^ Exploring the combination of immunomodulators with other anti-Mpox virus medications may be a promising avenue for further investigation.

### Modulation of the virus-induced cellular signal transduction

**Fig. 6:**
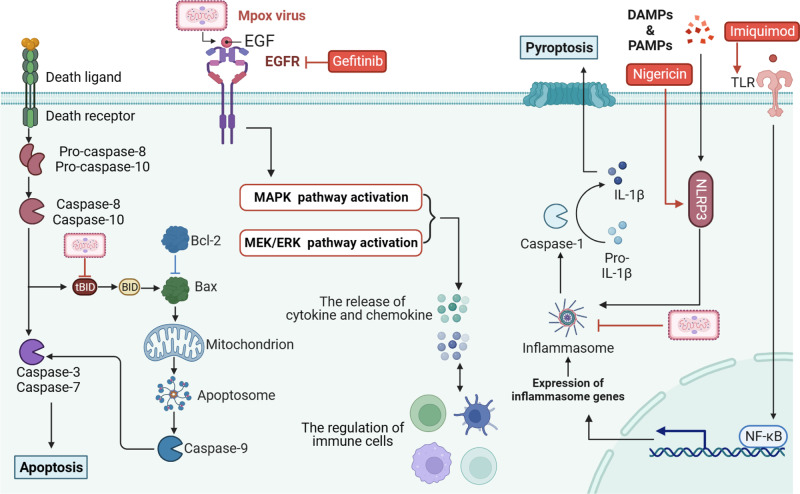
Illustrates the signaling pathways associated with the targeted actions of certain drugs following Mpox virus infection. Upon infection, Mpox inhibits pyroptosis, impeding the formation of inflammasomes and activation of caspase-1. This blockade prevents pyroptosis and hampers the adequate activation of the immune response against Mpox infection. However, nigericin, an activator of NLRP3 can induce pyroptosis in host cells, making it a promising candidate for an anti-Mpox drug. Moreover, tBID, a protein involved in apoptosis, is suppressed upon Mpox virus infection, thereby inhibiting both intrinsic and extrinsic apoptotic pathways and ensuring the survival of Mpox virus within host cells. This mechanism can be exploited by employing apoptosis inducers as a strategy to combat Mpox virus. Furthermore, Mpox virus infection triggers the binding of EGF and EGFR, activation downstream MAPK and MEK signaling pathways, leading to the release of inflammatory and chemotactic factors, and modulation of immune cells. That is, EGFR inhibitors like gefitinib may exhibit significant anti-Mpox activity

Mpox virus infection induces immune responses while also regulating cellular signal transduction.^215,216^ One example is the presence of a Mpox virus-encoded Bcl-2-like protein, which regulates the intrinsic apoptotic pathway. Additionally, the SPI-2 protein, encoded by the B12R gene,^217^ inhibits both caspase-1 and caspase-8, thereby disrupting the pyroptosis or apoptosis pathway,^218,219^ respectively. However, active induction of pyroptosis can be achieved by using nigericin, an inflammasome activator and pyroptosis inducer, as a strategy against Mpox infection. In an in vitro study conducted by Chad et al., Hela cells were infected with vaccinia virus and treated with Nigericin in vitro (VACV, EC_50_ = 7.9 nM, SI = 1038, in Hela cells).^220^ The findings demonstrated that Nigericin effectively reduced the viral titers and showed a stronger antiviral effect and lower EC_50_ values compared to the control group treated with Cidofovir. Protein kinases play a key role in regulating signal transduction pathways.^221,222^ Raghav et al. conducted an analysis to explore the interactions between Mpox virus and host proteins in order to further investigate the defense mechanisms triggered by Mpox infection. Their findings show the important role of the mitogen-activated protein kinase (MAPK) signaling pathway in the response to Mpox infection.^216^ Inhibition of the thymidine kinase enzyme, which is activated by MAPK, led to a significant reduction in viral replication.^223–225^ This evidence supports the potential of targeted therapies against MAPK signaling pathway as a promising strategy to combat Mpox (Fig. 6).^224,226^

## Prospect and challenges

With the global cessation of smallpox vaccination administration, the proportion of individuals with cross-immune protection against Mpox virus has rapidly declined, rendering Mpox a potential bioterrorism threat. While a few anti-Mpox drugs, such as Tecovirimat, have been clinically proven to be effective, relying solely on them would be unwise. Despite Mpox virus belonging to the DNA virus family, it exhibits significantly higher genomic variability due to increased nucleotide polymorphism. The rapid population mobility and increased international travel have facilitated the continuous spread of Mpox virus among populations, further increasing its potential for mutation.^11,227,228^ These factors contribute to increased variability, drug resistance, and the emergence of multidrug-resistant strains of Mpox virus.^229^ Moreover, currently available drugs face certain limitations that impede their clinical applications. For example, Cidofovir has low bioavailability and carries the risk of renal damage, while Cidofovir and Brincidofovir pose potential threats to hematopoietic and liver functions. There is an urgent need to develop novel anti-Mpox virus drugs.^230^

The lengthy and costly nature of drug development, combined with numerous uncertainties, has led to the exploration of drug repurposing strategies as a more efficient and economical approach.^110,231–233^ HTS of marketed drugs or clinically established medications has the potential to expedite the identification of antiviral agents, thus saving valuable time.^234–236^ For instance, the potential antiviral drug ribavirin has demonstrated therapeutic effectiveness against Mpox infection. Similarly, the widely used EGFR inhibitor gefitinib has shown promising antiviral activity against Mpox virus in addition to its approved indication for late-stage non-small cell lung cancer. However, drug repurposing efforts still heavily rely on serendipitous discoveries. Historically, drug development has been predominantly confined to laboratory settings. However, advances in computer science and computational drug design have significantly accelerated the discoveries in drug repurposing.^237–239^ Computer-aided drug discovery (CADD) techniques encompass the following three main directions: 1) High-throughput library screening of small molecule libraries, such as the discovery and development of Tecovirimat based on the VP37 protein. 2) Structural optimization based on existing drugs, such as NPP669, which involves alkyl chain modifications based on Cidofovir, resulting in overall improved pharmacological properties compared to Cidofovir. 3) Directly targeting functional sites for novel drug design, such as DdRp, which usually serves as a target for antiviral CADD.

In recent years, there has been relatively little attention on the Mpox outbreak in endemic area. However, the rapid spread of Mpox in non-endemic regions and its global impact have brought it back into the public attention. This particular outbreak of Mpox appears to exhibit distinct epidemiological characteristics and transmission dynamics compared to previous outbreaks.^63^ Previous knowledge suggested that the West African clade had weak transmission and pathogenicity compared to the Central African clade. However, the current situation reveals that the 2022-Mpox virus genome mutation and phylogenetic analysis indicate that this outbreak belongs to the B.1 lineage of the West African clade. The B1 lineage has exhibited mutations in virulence proteins, host recognition proteins, and immune evasion.^240^ APOBEC3 is an important enzyme that demonstrates antiviral activity against HIV, Hepatitis B Virus, Epstein-Barr virus, and other viruses through its functional cytidine deaminase activity.^241,242^ APOBEC3-mediated viral genome editing may be characterized by compatible substitutions GA>AA and TC>TT. Isidro et al. discovered a significant increase in G-to-A and C-to-T mutations in the recent Mpox isolates, with 46 SNPs showing mutation bias, among which 26 and 15 substitutions were GA>AA and TC>TT, respectively.^63,243,244^ These unusual mutation biases and the abundance of A: T bases in Mpox virus indicate that specific mutations driven by APOBEC3 may further reduce the pathogenicity and symptoms caused by Mpox virus infection, facilitating covert transmission within populations, and indirectly contributing to the global epidemic of Mpox. Although further experimental validation is necessary to confirm Mpox virus mutations mediated by APOBEC3, it is undeniable that APOBEC3 is a promising host antiviral protein for research. Understanding the mechanism of mutations driven by APOBEC3 may help reveal the mysteries behind the pathogenicity transition of Mpox.^245^ Additionally, the development of anti-Mpox drugs targeting APOBEC3 may provide a new direction for future drug development.

The development of multi-omics technologies and HTS techniques has enabled precise identification and characterization of various molecular targets of Mpox virus, which is crucial for the development of novel anti-Mpox virus drugs targeting new mechanisms. Furthermore, multi-omics technologies have revealed the gene expression patterns during Mpox infection and identified specific receptors and pathways regulated during Mpox progression. By precisely modulating these receptors and pathways, it is possible to develop drugs for Mpox therapy. This study contributes to optimizing the chemical structure of drugs, enhancing their delivery and targeting, thereby improving treatment precision and reducing drug side effects (Table 1).

**Table 1:** Anti-Mpox drugs and candidate compounds

	Name	Mechanism	Function	Clinical Use
Targeting virus intrusion	Amphotericin B	Isolate cholesterol and destroy lipid rafts	Restrict Mpox virus entry	☒
	Cholesterol lowering drugs			
	Glycosaminoglycan analog	Competitive binding to host cell membrane	Prevent the attachment and entry of Mpox virus	☒
Targeting virus replication	Cidofovir	Competitive binding of DNA or RNA polymerase	Interference with viral DNA or RNA synthesis	☑
	Brincidofovir			☑
	NPP-669			☒
	KAY-2–41			☒
	Trifluridine			☑
	Ribavirin			☑
	Ionomycin	Destroy the integrity of Endoplasmic reticulum	Inhibit the viral genome envelope formation	☒
Targeting virus assembly, maturation and release	Nocodazole	Promote microtubule depolymerization	Inhibit the movement of viral particles to the cell surface	☒
	Imatinib mesylate	Tyrosinase inhibition	Inhibit the actin tail formation	☒
	PA104	Inhibit the actin tail formation	Inhibit Mpox virus efflux	☒
	Tecovirimat	Inhibit vp37 protein synthesis	Inhibit the maturation and budding release of orthopoxviruses	☑
	NIOCH-14			☒
	Gefitinib	EGFR inhibition	Inhibit the actin tail formation	☒
	MEK inhibitors	MEK inhibition	Inhibit the actin tail formation	☒
	A36R polypeptide	Anti Mpox virus-A36R	Inhibit Mpox virus transmission and release	☒
Immunoregulation	VIGIV	antigen binding	Prevent Mpox virus infection of target cells	☑
	Pidotimod	Enhancing specific and non-specific immunity	Enhance immune response	☒
	Thymopeptide			☒
	mAbs	Destroy virus particles	Prevent virus infection of cells	☒
	Nigericin	Activate IL-1β and IL-18	Induced pyroptosis	☒
	Imiquimod	TLR agonists and local immune activity enhancer	Stimulate cytokine production and activate local immunity	☒

In recent years, AI, especially machine learning and deep learning methods, have increasingly been utilized in various stages of the drug development process, challenging the traditional paradigms of new drug discovery and design.^246^ By leveraging extensive compound data within libraries, AI enables efficient design and optimization of compounds targeting various Mpox virus homologous proteins. This approach facilitates the effective screening of optimal anti-Mpox virus drugs or the development of promising new candidate molecules, ultimately reducing the cost and time associated with drug development.^247,248^ However, it is important to note that no commercially available drugs have emerged from this approach yet, indicating the need for further technical advancements and breakthroughs in the field.^249^

In addition to the development of systemic anti-infective drugs, exploring local therapies for Mpox is crucial. Mpox infections can cause severe physiological and psychological trauma to the skin and eyes.^250^ This damage is often visibly evident and difficult to conceal. Skin lesions, for example, are a hallmark of Mpox infection and inflict immense pain on patients. The psychological trauma resulting from these skin injuries and subsequent scarring may surpass the physical harm. A distressing incident reported in 2017 by Dimie et al. highlighted the tragic suicide of a 34-year-old Mpox patient due to the psychological trauma endured post-infection.^251^ Therefore, addressing skin lesions during the course of Mpox infection is essential. Notably, the topical cream imiquimod has demonstrated particular efficacy in treating Mpox-induced skin lesions.^252,253^ The exact mechanism of action of imiquimod in the treatment of Mpox infections is not fully understood, although several potential mechanisms have been proposed. Imiquimod acts as an agonist for Toll-like receptor 7 (TLR-7) and Toll-like receptor 8 (TLR-8), triggering the nuclear translocation and transcriptional activity of nuclear factor κB (NF-κB). This activation leads to the release of downstream pro-inflammatory cytokines, enhancing the immune response against Mpox. Additionally, imiquimod acts as a direct local immune stimulant by stimulating the production of various cytokines, including IFN-γ, TNF-α, IL-1β, and IL-6. These cytokines play a crucial role in activating the innate immune system and promoting a localized immune response.^254–256^ Studies have shown that imiquimod can recruit plasmacytoid dendritic cells to the site of infection, thereby enhancing the antigen presentation process. Although ocular infections caused by Mpox are relatively rare, they may result in permanent visual impairment, including irreversible conditions such as corneal perforation. As a locally administered nucleoside analog, trifluridine eye drops are currently considered the most effective treatment for ocular Mpox infections. In addition to controlling Mpox proliferation in the eyes, trifluridine eye drops also help reduce the production of conjunctival secretions, thereby minimizing the risk of spreading the infection to others.

To address the widespread occurrence of Mpox, it is imperative to recognize it as a global public health concern. In addition to the development of therapeutic medications, emphasis must be placed on preventive measures. Prevention, especially among areas with active Mpox transmission and those in high-risk populations like HIV-infected MSM, is crucial.^257^ Vaccination is an effective strategy for preventing Mpox. Studies indicate indicates that vaccinia vaccine can offer partial protection against Mpox infection.^258,259^ However, the use of smallpox vaccines for Mpox prevention in epidemic regions limited due to potential risks for immunocompromised individuals, particularly those co-infected with HIV. First-generation vaccines like Dryvax and second-generation vaccines such as ACAM2000, which are live replicating vaccinia virus vaccines, can cause severe infections such as progressive vaccinia.^260^ The emergence of third-generation vaccines provides an alternative for this specific population. One example is Imvamune (also known as JYNNEOS) a non-replicating vaccinia vaccine that has been tested safe for HIV-infected patients. Animal models have shown that JYNNEOS also provides protection against Mpox.^261^ In 2019, the FDA approved JYNNEOS for preventing Mpox infection in high-risk populations aged 18 and above.^262^ Clinical evidence has demonstrated that JYNNEOS vaccination effectively prevents Mpox cases and reduce the incidence of severe illness.^258,263,264^ At the national and regional levels, enhancing public health investments, including environmental sanitation and disinfection, and establishing efficient case identification and contact tracing mechanisms, is essential. On an individual level, it is crucial to educate oneself about Mpox, maintain good personal hygiene, employ personal protective measures, and avoid contact with infection sources. Therefore, the most cost-effective method to reduce the incidence and transmission of Mpox is through implementing preventive measures rather than solely relying on the development of novel anti-Mpox drugs.

## Conclusions

While some progress has been made in the development of drugs against Mpox, it is crucial to expedite the research progress. This will enable us to effectively combat potential long-term outbreaks and the emergence of drug-resistant Mpox virus strains. In the development of drugs against Mpox, the following aspects should be given priority: Firstly, improving the specificity and delivery efficiency of drugs is essential to ensure accurate targeting of the Mpox and efficient transmission to the infection site. Secondly, development anti-Mpox drugs that are less prone to resistance is necessary to prevent the gradual emergence of drug-resistant strains and ensuring sustained efficacy of treatment. Additionally, exploring the development of sequential and combination drug therapies should enhance effectiveness against different stages of Mpox infections and their variants. Lastly, attention should be paid to drug modifications to mitigate or eliminate toxicity, minimizing the adverse impact on patients during the treatment process. The early investment in drug development against Mpox is crucial in tackling the ongoing global Mpox outbreak. Accelerating progress in the development of effective anti-Mpox drugs will help prepare for future challenges and provide more reliable protection for public health.
